# Synaptic Basis for the Generation of Response Variation in Auditory Cortex

**DOI:** 10.1038/srep31024

**Published:** 2016-08-03

**Authors:** Can Tao, Guangwei Zhang, Chang Zhou, Lijuan Wang, Sumei Yan, Li I. Zhang, Yi Zhou, Ying Xiong

**Affiliations:** 1 Department of Neurobiology, College of Basic Medical Sciences, Third Military Medical University, 30 Gaotanyan St., Chongqing, 400038, China; 2 Zilkha Neurogenetic Institute, Keck School of Medicine, University of Southern California, Los Angeles, California 90033, USA

## Abstract

Cortical neurons can exhibit significant variation in their responses to the same sensory stimuli, as reflected by the reliability and temporal precision of spikes. However the synaptic mechanism underlying response variation still remains unclear. Here, *in vivo* whole-cell patch-clamp recording of excitatory neurons revealed variation in the amplitudes as well as the temporal profiles of excitatory and inhibitory synaptic inputs evoked by the same sound stimuli in layer 4 of the rat primary auditory cortex. Synaptic inputs were reliably induced by repetitive stimulation, although with large variation in amplitude. The variation in the amplitude of excitation was much higher than that of inhibition. In addition, the temporal jitter of the synaptic onset latency was much smaller than the jitter of spike response. We further demonstrated that the amplitude variation of excitatory inputs can largely account for the spike variation, while the jitter in spike timing can be primarily attributed to the temporal variation of excitatory inputs. Furthermore, the spike reliability of excitatory but not inhibitory neurons is dependent on tone frequency. Our results thus revealed an inherent cortical synaptic contribution for the generation of variation in the spike responses of auditory cortical neurons.

Neural information is coded in the form of spiking activity, yet the spike responses of a neuron to the same stimuli are not consistent: spike response can fail in some trials and spike latency can be variable. Variation in spike responses is broadly found throughout the sensory system^1^,^2^ and is considered as an important factor for reliable sensory information coding, but the mechanism underlying spike variation is not well understood. Fluctuation of intrinsic factors like threshold and resting membrane potential have been proposed as possible sources^3^,^4^, but were found insufficient to explain the spike variation^5^,^6^,^7^. Recent work found that manipulation of presynaptic neurons can alter spike variation^8^, suggesting the synaptic inputs are significant for the response variation.

Cortical neurons receive both excitatory and inhibitory synaptic inputs and the interplay between the two components predominantly determines spike responses in the sensory cortex^9^,^10^,^11^. Despite some modeling work^12^, little is known about the contribution of different synaptic inputs to the variation of spike responses. Do cortical neurons receive relatively stable or highly variable excitatory/inhibitory synaptic inputs from presynaptic neurons and how much variation observed in spike responses can be credited to fluctuating synaptic transmission? Direct measurement and systematical evaluation of different synaptic inputs are essential to reveal the synaptic mechanism in the generation of spike variation.

In this study, we first confirmed and characterized the variation of single neuron activities in layer 4 of rat primary auditory cortex(A1) *in vivo*. Second, to determine the contribution of threshold, resting membrane potential and postsynaptic potential evoked by sensory stimuli, membrane potential responses were recorded and evoked post-synaptic potentials are found to be most significant in the generation of variation. To dissect the roles of excitatory and inhibitory synaptic inputs in the variation of post-synaptic potential, *in vivo* whole cell voltage-clamp recordings were performed. Synaptic inputs were reliably induced by repetitive stimulation, but with relatively large variation in amplitude. The variation in the amplitude of excitation was much higher than that of inhibition. In addition, the temporal jitter of the synaptic onset latency was much smaller than the jitter of integration time. We further demonstrated that the amplitude variation of excitatory inputs can largely account for the spike variation, while the jitter in spike timing can be primarily attributed to the temporal variation of excitatory inputs. Furthermore, the spike reliability of excitatory but not inhibitory neurons is dependent on tone frequency. Our results provide direct experimental evidence that the response variation of auditory cortical neurons is primarily due to variation in afferent excitation.

## Results

### Spiking reliability and temporal precision of excitatory neurons in A1

**Figure 1:**
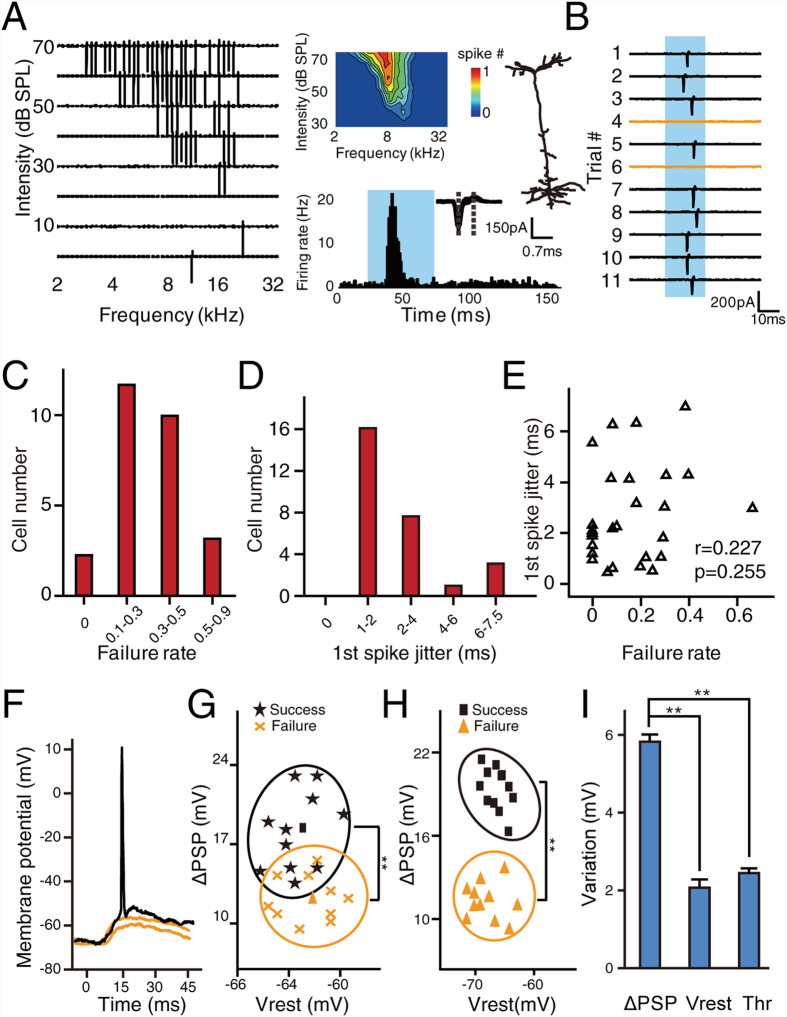
Supra- and subthreshold spike variation of excitatory neurons in A1. (**A**) A representative TRF recorded from an excitatory neuron in A1. Left, raw spike TRF from one trial. Each small trace in the TRF represents the evoked spike response (50 ms window) to a pure tone. Right, TRF of the averaged spike response and cell morphology as reconstructed by biocytin histology (upper), peristimulus time histogram (lower, bin = 1 ms), and superimposed spike waveforms (inset, n = 50, randomly chosen). The shaded area indicates the presence of the pure tones. The dashed lines indicate the trough-to-peak interval. (**B**) Spike responses to pure tones at CF (20 dB above threshold) that were recorded over 11 repetitions. The yellow line indicates trials failed to generate spikes. The shaded area indicates the presence of stimuli. (**C**) The distribution of response FR to repetitive pure tones at CF (20 dB above threshold) from 27 excitatory neurons. (**D**) The distribution of 1st spike jitter to repetitive pure tones at CF (20 dB above threshold) from 27 excitatory neurons. (**E**) Scatter plot of FR versus 1st spike jitter (n = 27 neurons). r, Pearson correlation coefficient. p, correlation significance. (**F**) Three examples of membrane potential response to repetitive pure tones (CF, 20 dB above threshold). The yellow line indicates trials failed to generate spikes. The stimulation starts at 0 ms with a duration of 35 ms. (**G**) Scatter plot of ΔPSP versus resting membrane potential for responses with and without spikes (21 trials). The clustering is based on spike responses. Black cluster, successful spike; Yellow cluster, failed spike. The square and triangle indicate the average value for successful spikes and failed spikes, respectively. **p<0.01, *t*-test. (**H**) Scatter plot of the average ΔPSP versus the average resting membrane potential from 11 recorded excitatory neurons. The ΔPSP and resting membrane potential were grouped based on whether the spike response was successful or failed. **p < 0.01, paired *t*-test. (**I**) Comparison between the variation of ΔPSP, resting membrane potential(Vrest) and threshold(Thr). n=11, **p < 0.01, one-way ANOVA and post hoc test. Error bar, S.E.M.

A short pure-tone pulse evokes only one or no spikes in layer 4 excitatory neurons in A1 under anesthetized conditions, and this phenomenon has been termed “binary coding”^11^,^13^. Due to the binary nature of the spiking responses, the response variation in A1 can be quantified by measuring the reliability and temporal precision of the evoked spike. To measure reliability, failure rate (FR) was defined as the ratio between the number of trials that failed to generate a spike and the total number of trials. To measure temporal precision, 1st spike jitter was calculated as the standard deviation of the onset latencies of evoked spikes across trials. Figure 1A shows the spiking tonal receptive field (TRF) of a representative excitatory pyramidal cell recorded by loose-patch recording (see Materials and Methods). Figure 1B shows the raw traces of 11 recorded trials in which a preferred stimulus was given (at the characteristic frequency, or CF, and at 20 dB above threshold), of which 2 trials failed to evoke spikes. As different neurons might have different sensitivity, the sound pressure level of sound stimuli was set at a moderate level (20 db above threshold) based on the sensitivity of each recorded neuron, which has been widely used in auditory studies^11^,^14^. To quantify the variation in spike activity at CF, the FR and 1st spike jitter were calculated (see Materials and Methods). Figure 1C,D shows the distribution of spike FR and jitter of 27 excitatory pyramidal neurons (20 dB above threshold, CF). Most neurons exhibited a significant FR in their responses to repetitive CF tones at a moderate intensity. Relatively poor reliability (FR > 0.3) was observed in 44% of the recorded neurons. We then investigated the temporal precision of the spike responses (Fig. 1D). A notable number of neurons (40.7%, 11 of 27) showed relatively large jitter in their first spike responses, i.e., jitter > 2 ms. The 1st spike jitter could be as large as 7.5 ms. No strong correlation was found between FR and 1st spike jitter (r = 0.227, *p* = 0.255, Fig. 1E). It also worth noting that for excitatory neurons, the variability changes as sound level increases (Fig. S1A,B).

To understand the role of intrinsic properties (e.g. resting membrane potential, spike threshold) and the synaptic inputs evoked by sensory stimulation in the generation of spike variation, the *in vivo* current-clamp technique was used to record membrane potential changes. Figure 1F shows 3 representative trials of membrane potential changes evoked by CF tone (20 dB above threshold). Although spike responses to repetitive CF tone might occasionally fail, subthreshold responses were consistently observed from all recorded neurons (n = 13). This result suggested that “failed” spikes were not due to “failed” synaptic inputs (no synaptic input at all). We then measured the amplitudes of ΔPSP and resting membrane potential (Vrest), and grouped them into two clusters according to the presence of spikes. As shown in Fig. 1G, for an excitatory neuron, when the responses were evoked by repetitive stimuli (CF, 20 dB above threshold, 21 trials), the ΔPSPs in cases with successful spiking were significantly larger than those in cases of failure (p < 0.01, *t*-test). In contrast, no significant difference was observed for Vrest between groups. The same conclusion was obtained for 11 excitatory neurons (p < 0.01, paired *t*-test, Fig. 1H). The spike threshold was also found variable (−52.24 ± 2.56 mV, mean ± S.D.), but the variation of ΔPSP(S.D. = 6.08 mV) is significantly larger than that of resting membrane potential(S.D. = 2.15 mV) and spike threshold(S.D. = 2.56 mV) (n = 11, p < 0.01, one-way ANOVA with post hoc test, Fig. 1I). These results suggested that for the excitatory neurons recorded in A1, the spike failure was largely determined by the amplitude fluctuation of ΔPSP evoked by sensory stimuli.

### Variation in synaptic inputs to excitatory pyramidal neurons

**Figure 2:**
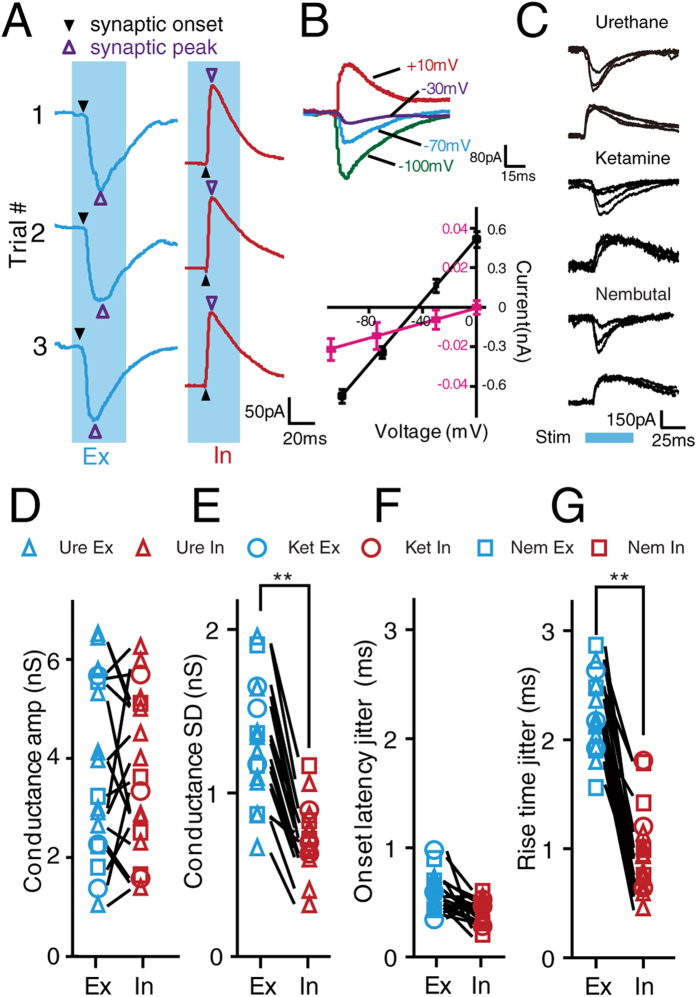
*In vivo* voltage-clamp recording of excitatory and inhibitory synaptic inputs. (**A**) Example of excitatory (blue) and inhibitory (red) synaptic responses to repetitive pure tones (CF, 20 dB above threshold, 3 trials). The solid and open triangles indicate the onset and peak of the synaptic inputs, respectively. The shaded area indicates the presence of the pure tones. (**B**) Synaptic responses to white noise (60 dB) recorded at different holding potentials (Upper, −100, −70, −30, and +10 mV). Lower, I-V curves for synaptic currents averaged from 5 repeats. The curves were generated using values from within 0–1 ms (red) and 20–22 ms (black) windows after the onset of responses. Error bar, SEM. (**C**) Demonstration of excitatory and inhibitory synaptic inputs recorded under different anesthetic drugs. Shaded area indicates the presence of pure tone. (**D,E**) Comparison of the average amplitude (**D**) and standard deviation of amplitude (**E**) of excitatory and inhibitory conductance from 10 excitatory neurons. Data recorded from the same neuron are linked with a line. **p < 0.01, paired *t*-test. (**F,G**) Comparison of the onset latency jitter (**F**) and rise time jitter (**G**) of excitatory and inhibitory synaptic inputs. Data recorded from the same neuron are linked with a line. **p < 0.01, paired *t*-test. The combination of color (blue and red) and shape (triangle, circle, and square) indicate the excitatory and inhibitory synaptic inputs that were recorded under different anesthetic conditions. The statistical test were conducted using the data collected under urethane anesthesia (urethane, n = 10; ketamine+xylazine, n = 3; Nembutal, n = 4).

The membrane potential responses of an auditory cortical neuron are primarily determined by the integration of sound-evoked synaptic excitation and inhibition^15^,^16^. To dissect the respective roles of excitation and inhibition in the generation of spike variation, *in vivo* whole-cell voltage-clamp recording was employed (see Materials and Methods, Fig. 2A). To record tone-evoked synaptic conductance, neuronal membrane potentials were clamped at −70 mV and +10 mV to isolate excitatory and inhibitory currents, respectively. The quality of voltage-clamp in our recordings was reasonably good, as demonstrated by the linear I-V relationship of the recorded synaptic currents (Fig. 2B). Because the effects of anesthesia may alter synaptic inputs, we also tested the variation in synaptic inputs under the influence of different anesthetic drugs (urethane: n = 10, ketamine + xylazine mixture: n = 3 and Nembutal: n = 4, see Materials and Methods for details, Fig. 2C). While no significant differences were found between the averaged conductance of excitatory and inhibitory inputs (Fig. 2D), the variation in the conductance of excitatory inputs (measured by S.D.) was significantly larger than that of inhibitory inputs (Fig. 2E) and this change was similar in all drug conditions. And the coefficient of variation of excitation (0.33 ± 0.1) is also significantly larger (p < 0.01, paired-*t* test) than that of inhibition (0.15 ± 0.07), further supporting our observation of a more variable excitation rather than inhibition.

Next, we examined the temporal precision of excitatory and inhibitory synaptic inputs. The onset latency of synaptic input was defined as the interval between stimulation onset and the starting point of the evoked synaptic input. Variation in the synaptic onset latency can reflect the temporal precision of thalamocortical inputs. The onset latency of excitatory synaptic input (11.12 ± 0.61 ms) was shorter (2.11 ± 0.72 ms) than that of inhibitory input (13.23 ± 0.47 ms), supporting a disynaptic nature of inhibition from the auditory thalamus as previously reported^17^. It was also notable that the onset latency jitter of both excitatory (0.61 ± 0.10 ms) and inhibitory inputs (0.47 ± 0.08 ms) was very small, suggesting a highly precise arrival time of synaptic input (Fig. 2F). In addition, the rise time of different synaptic inputs that were recorded from the same neuron were also compared (Fig. 2G). The rise time was measured as the interval between the onset and the peak of the evoked synaptic input and can reflect the temporal profile of synaptic currents. The variation in the rise time of excitatory inputs (2.61 ± 0.34 ms) was significantly larger (p < 0.001, paired *t*-test) than that of inhibitory inputs (1.02 ± 0.29 ms). Considering that the amplitude of excitatory inputs also fluctuates more than that of inhibitory inputs, these results suggested that the excitatory synaptic inputs could be the major contributor of variation in synaptic integration.

### Synaptic contribution to spike reliability

The experimental data clearly demonstrated variation in the excitatory and inhibitory synaptic inputs, yet it is difficult to interpret the contributions of different variables (e.g., amplitude, temporal jitter) and different synaptic inputs to the generation of spike variation. For example, will equal degrees of variation in the amplitude of excitatory inputs and inhibitory inputs result in similar changes in spike variation? How much spike variation will be generated by synaptic inputs with variable amplitudes and stable temporal profiles or by inputs with stable amplitudes and variable temporal profiles? These questions cannot be easily answered with physiological data alone. Besides, QX-314 and cesium chloride added in the internal solution which block Na^+^ and K^+^ channels can improve the recording quality, but also make it impossible to obtain spiking responses from neurons under whole-cell voltage-clamp recording. Thus, to find possible answers to these questions, we exploited an integrate-and-fire model^11^ for evaluation. In the Supplementary Figure S2, we estimated the variation generated by different fluctuation in synaptic excitation and inhibition. The model suggested that driving force of excitatory synaptic input is more prominent than that of inhibitory synaptic input. Thus, fluctuated excitation can be dominant in the generation of spike variation (Fig. S2E,F). In addition, the effect of excitation variation on membrane potential is amplitude dependent. When the synaptic excitation is relatively strong, fluctuation of excitatory synaptic input can reduce the spike reliability; on the other hand, while amplitude of synaptic input is relatively weak; excitatory fluctuation can even increase the spike reliability (Fig. S2G,H, see Supplementary materials for details).

### Generation of temporal jitter in spike responses

**Figure 3:**
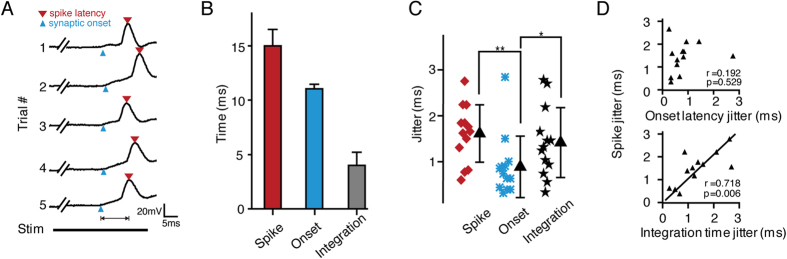
Generation of temporal jitter in spike responses. (**A**) Five trials of membrane potential responses to stimulation with the same tone (CF, 20 dB above threshold). The synaptic onset latency and spike latency are marked with blue and red triangles, respectively. The thick black double-line indicates the presence of sound stimulation. The line with arrowheads between the onset of synaptic input and the spike indicates the integration time. (**B**) Comparison of the 1st spike latency (Spike), onset latency (Onset) and integration time (Integration) of the neuron shown in (**A**), Error bar, S.D. (**C**) Scatter plot of 1st spike jitter, onset latency jitter and integration time jitter from 13 recorded neurons. *p < 0.05, **p <0.001, One-way ANOVA and *post hoc* test. (**D**) Scatter plots of onset latency jitter and integration time jitter versus 1st spike jitter.

The temporal precision of spike activity was proposed to be important for normal brain function^5^. Temporal jitter in auditory cortex could be attributed to two components^16^: the temporal variation relayed from the thalamocortical projection (onset latency) and the temporal variation generated de novo by synaptic integration (integration time). *In vivo* current-clamp recording was employed to measure the temporal details of both suprathreshold and subthreshold responses simultaneously. Figure 3A shows the membrane potential changes recorded from an excitatory neuron during 5 trials of the presentation of the CF tone (20 dB above threshold). Two important temporal parameters of the membrane potential changes were quantified: the onset of depolarization and the integration time. Depolarization onset indicates the arrival of synaptic input, and the integration time reflects the time spent on spike integration. The integration time was measured as the interval between depolarization onset and the timing of spikes. Figure 3B shows the spike times (14.82 ± 1.93 ms), onset latency (11.03 ± 0.32 ms) and integration time (4.32 ± 1.78 ms) for 10 trials from the representative neuron shown in Fig. 3A. The temporal jitter in the onset latency of synaptic inputs is much smaller than the jitter in the spike responses and integration time. Statistical results from 13 excitatory pyramidal neurons further confirmed that (Fig. 3C). This result suggested that during cortical computation, temporal imprecision was actually increased by the highly fluctuating integration time. To quantify the relationship between jitters of onset latency, integration time and 1st spike latency, we calculated the Pearson correlation coefficients between these parameters (Fig. 3D). The Pearson correlation analysis showed strong correlation between jitters of integration time and 1st spike latency (r = 0.718, *p* < 0.05, n = 13) but not between jitters of onset latency and 1st spike latency (r = 0.192, *p* = 0.529, n = 13). These results suggested that the temporal precision of thalamocortical inputs is reliable and that the temporal variation in spike responses can be mainly attributed to variation in the integration time. To better understand the role of evoked synaptic inputs in the generation of temporal jitter, we further examined the contribution of fluctuating excitatory and inhibitory synaptic inputs to spike precision using the integrate-and-fire model. Modeling results further confirmed that the temporal jitter in spike responses could be largely attributed to fluctuation in temporal profiles of the excitatory inputs and that stable inhibitory inputs could help to reduce the temporal variation (see Fig. S3 and Supplementary materials for details).

### Spike reliability of excitatory neurons is frequency dependent

**Figure 4:**
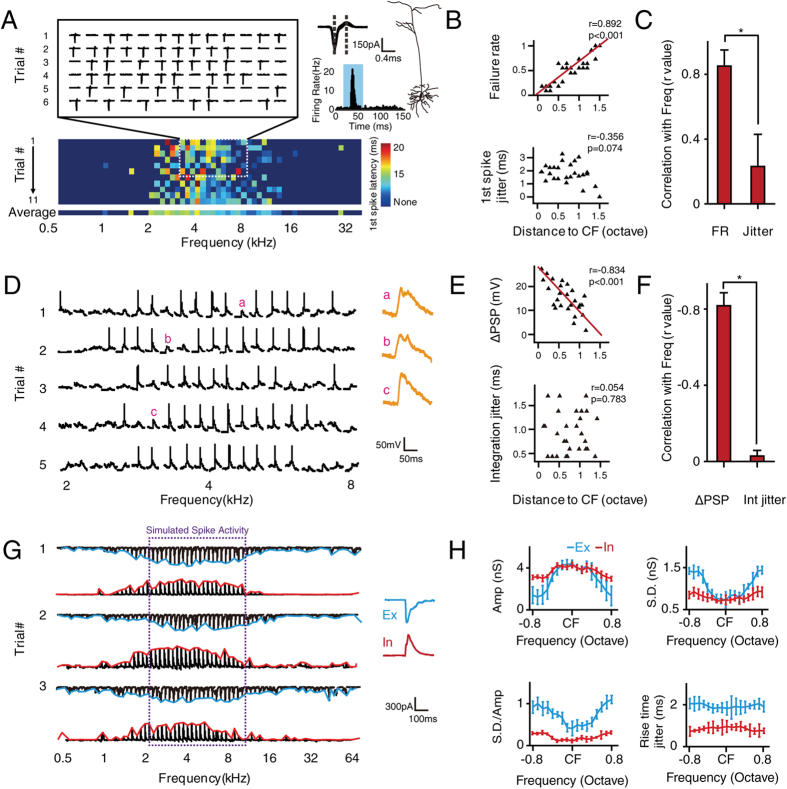
Synaptic mechanisms underlying the frequency selectivity of spike variation. (**A**) Upper left, six trials of representative spike responses to pure tones within 1.3 octaves of CF (20 dB above threshold). Upper right, superimposed spike waveforms (n = 50, randomly chosen), cell morphology as reconstructed by biocytin histology, and the peristimulus time histogram of the recorded neuron (bin = 1 ms). Lower, 11 trials of spike responses to pure tones of various frequencies represented by first spike latency. Deep blue indicates no spike. The shaded area indicates the presence of stimuli. Dashed lines indicate the trough-to-peak interval. (**B**) Upper, scatter plot of the distance to CF versus FR. Lower, scatter plot of the distance to CF versus 1st spike jitter, based on statistics from the representative neuron shown in (**A**). (**C**) Pearson correlation coefficient measured between the FR, 1st spike jitter and the relative distance to CF (n = 12 neurons). Jitter, 1st spike jitter. Bar, S.D. *p = 0.0117, using Fisher’s Z-transformation. (**D**) Left, five trials of representative membrane potential responses to pure tones of various frequencies (20 dB above threshold). Right, three enlarged responses without spike. The scale bar is for traces on the left side. (**E**) Upper, scatter plot of the distance to CF versus ΔPSP. Lower, scatter plot of the distance to CF versus integration time jitter, based on statistics from the representative neuron shown in (**D**). (**F**) Pearson correlation coefficient measured between the ΔPSP, integration time jitter and the relative distance to CF (n = 7 neurons). Int jitter, integration time jitter. Bar, S.D. *p = 0.0409, using Fisher’s Z-transformation. (**G**) Left, three trials of representative excitatory and inhibitory responses to pure tones of various frequencies (20 dB above threshold). Right, averaged synaptic response to the CF. In, inhibitory input; Ex, excitatory input. The purple dashed lines show the region of simulated spike activity. (**H**) Frequency tuning of the excitatory and inhibitory conductance(Amp), variation of the conductance (S.D.), ratio between the variation and the amplitude(S.D./Amp) as well as the synaptic rise time jitter (n = 9). In, inhibitory conductance; Ex, excitatory conductance.

We have demonstrated and analyzed the role of synaptic inputs in the generation of response variation based on the results of repetitive pure tones at CF with moderate level (20 dB above threshold), where the synaptic inputs should be relatively strong and robust. Yet the results showed that even at preferred frequency, the spikes responses and synaptic inputs are still highly variable in pyramidal cells (especially the excitation). It remained unclear if the variation of synaptic input is consistent when non-preferred stimuli is given (away from CF). By further presenting tone stimuli of different frequencies (0.5–64 kHz, 0.1 octave step, 20 dB above threshold, Fig. 4A), we found that there was a strong correlation between FR and frequency (r = 0.892, p < 0.001, Fig. 4B). Meanwhile there was not a strong correlation between 1st spike jitter and frequency (r = −0.356, *p* = 0.074, Fig. 4B). Statistical results from 12 neurons showed that FR was correlated with sound frequency (r = 0.89 ± 0.12, Fig. 4C) but not with 1st spike jitter (r = 0.23 ± 0.21, Fig. 4C). In addition, as shown by *in vivo* current-clamp recordings (Fig. 4D), within the receptive field, “failed spikes” were still consistently associated with subthreshold activity, even at non-CF frequencies (Fig. 4D). These observations further confirmed our previous results (Fig. 1F). A strong correlation was also found between the ΔPSP and sound frequency but not between integration time jitter and frequency (Fig. 4E). This has been further validated by statistical results from 7 neurons (Fig. 4F).

To better understand the synaptic basis of the frequency selectivity of spike reliability, both the excitatory and inhibitory synaptic inputs evoked by the pure tones with different frequencies were recorded from 10 excitatory pyramidal neurons. Figure 4G shows three pairs of representative excitatory and inhibitory responses recorded from one neuron, and Fig. 4H shows the tuning curve of the excitatory and inhibitory conductance versus frequency. The excitatory inputs were frequency selective, whereas the inhibitory inputs were more broadly tuned. The variation (S.D.) in the amplitude of excitation was also best tuned to the preferred frequency. The average data from 10 neurons also showed similar results (Fig. 4H). Thus, the frequency dependence of the spike variation could be explained by the frequency selectivity of the amplitude and temporal variation of excitatory and inhibitory synaptic inputs.

### Inhibitory neurons in A1 exhibit highly reliable spike responses

**Figure 5:**
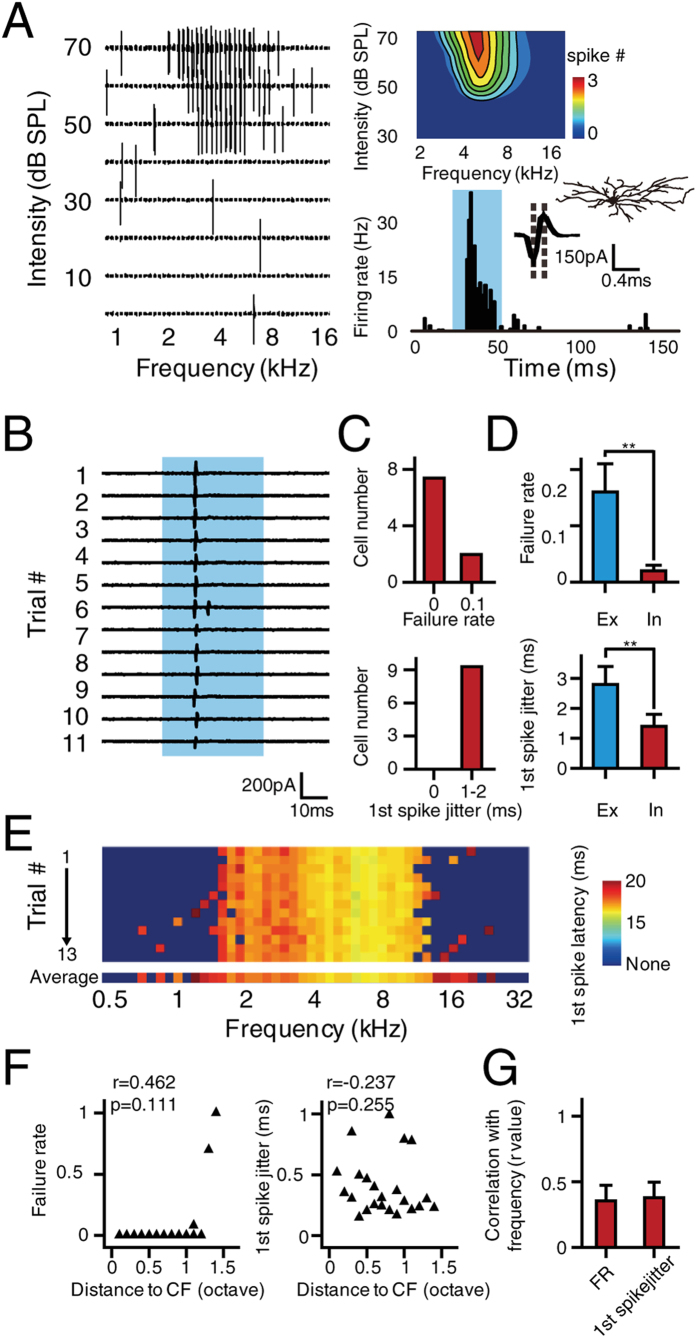
Spike variation of inhibitory interneurons. (**A**) A representative TRF recorded from an inhibitory interneuron in A1. Left, raw spike TRF from one trial. Each small trace in the TRF represents the spike response (50 ms window) evoked by a pure tone. Right, TRF of the averaged spike response (upper), peristimulus time histogram (lower), and superimposed spike waveforms (inset). The shaded area indicates the presence of the pure tones. Dashed lines indicate the trough-to-peak interval. The cell morphology, as reconstructed by biocytin histology, is shown in the corner. (**B**) Spike responses to the presentation of a pure tone at the CF (20 dB above threshold) that were recorded from eleven repetitions. The shaded area indicates the presence of the pure tones. (**C**) The distribution of response FR (upper) and 1st spike jitter (lower) to repetitive pure tone stimulation at the CF (20 dB above threshold) from 9 inhibitory interneurons. (**D**) Comparison of FR (upper) and 1st spike jitter (lower) between excitatory and inhibitory neurons. **p < 0.001. Excitatory neurons, n = 27, inhibitory neurons, n = 9. (**E**) Thirteen trials of complete spike responses to pure tones (0.5–32 kHz) represented by the latency of the first evoked spike. (**F**) Scatter plot of the distance to CF versus FR (Left) and 1st spike jitter (Right), based on statistics from the representative neuron shown in (**E**). (**G**) Pearson correlation coefficient measured between the FR, 1st spike jitter and the relative distance to CF from 9 inhibitory interneurons. Bar, S.D. *p* = 0.484, using Fisher’s Z-transformation.

*In vivo* whole-cell voltage-clamp recording from pyramidal neurons have suggested that inhibitory synaptic conductance are much more stable than excitation with respect to both amplitude and temporal profile (Fig. 2C–E). As intracortical inhibition is mainly contributed by local inhibitory interneurons^18^,^19^,^20^, we applied *in vivo* loose-patch recording to study the variation in the spiking of fast-spiking inhibitory neurons specifically to further understand the origin of the stable inhibition. Figure 5A shows an example TRF of an inhibitory interneuron recorded in A1, and Fig. 5B presents 11 trials of spike responses to repetitive pure tone stimulations at the CF (20 dB above threshold). In contrast to those of the excitatory neurons, (Fig. 1B), the spike responses of the inhibitory neuron to repetitive tone stimulations was very stable (Fig. 5B), as reflected by a low FR (Fig. 5C, upper panel) as well as small 1st spike jitter (Fig. 5C, lower panel). We further compared the average FR and 1st spike jitter between excitatory neurons and inhibitory neurons. We found that both the reliability and temporal precision of spiking were significantly higher in the inhibitory neurons than in the excitatory neurons (both p < 0.001, Fig. 5D). We also examined the frequency selectivity of the reliability and temporal precision of spiking in the inhibitory neurons. Even if the stimulation frequency was away from the CF, the spike responses of the inhibitory neurons were still highly reliable (Fig. 5E). More interestingly, neither the FR nor the 1st spike jitter of the inhibitory neurons showed strong frequency dependence (Fig. 5F), which is different from the frequency dependence found in the excitatory neurons (Fig. 4C). Statistical results based on correlation analysis also confirmed that the variation in the spiking of the inhibitory neurons was not frequency selective (Fig. 5G). Taken together, these results suggested that cortical inhibitory neurons exhibit highly reliable responses to repetitive stimulation and that cortical variation can be primarily attributed to variation in the responses of excitatory neurons. In addition, the poor frequency selectivity of the inhibitory neurons could also be due to their highly reliable responses to all frequencies within the receptive field.

## Discussion

It remains unclear whether variability in neural response is beneficial to information coding (e.g., stochastic resonance) or is merely biological noise^6^,^21^. Nevertheless, almost any neuroscience study requires enough repetitions to obtain a trustworthy result based on averaging. The scope of the study is to investigate whether and how the excitatory and inhibitory synaptic interaction can contribute to the response variation of the auditory cortical neurons. In the present study, characterization of spike variation revealed that the sound-evoked spike responses of excitatory neurons in the auditory cortex can exhibit relatively large variation, which could be largely attributed to fluctuation in excitatory synaptic inputs. On the other hand, the cortical inhibition generated by inhibitory neurons was found to be reliable with respect to both amplitude and temporal profile. Because the cortex does have the capability to generate highly reliable responses, the variation of responses in excitatory neurons could be a strategy for energy efficiency in information coding.

Response variation exists from the perception of sensory signals to the behavior of animal; thus, response variation poses a fundamental problem for information processing^22^. In layer 4 of the auditory cortex, variation in neural responses reflects the overall result of fluctuation relayed from earlier stages (e.g., from the medial geniculate body) and generated *de novo* by cortical circuits. Our results suggested that besides the inherited variation, local integration of synaptic inputs is also an important source of response variation in auditory cortex. The interplay between excitatory and inhibitory synaptic inputs can not only control the spike reliability but also contribute to the temporal jitter of spike responses.

By performing whole-cell current-clamp and voltage-clamp recordings of excitatory neurons, we determined the change in amplitude as well as the temporal precision of both excitatory and inhibitory synaptic inputs evoked by repetitive pure tone. We found that compared to the consistent FR that was observed in the spike responses, both subthreshold membrane potential depolarization and evoked synaptic inputs could be robustly observed. This result supports the hypothesis that a failed spike does not necessarily come from a total failure in synaptic input. Again, it is the synaptic integration of intrinsic dynamics and different synaptic components that finally decides if a spike will be generated. In addition, the fluctuation of excitation is the leading factor in the generation of spike variation, including both the FR and temporal jitter.

In this study, we found that the spike reliability of inhibitory neurons is much higher than that of excitatory pyramidal cells. The inhibitory neurons could faithfully respond to repetitive stimuli, indicating that the response reliability of cortical neurons should not be limited by the nature of biological fluctuations. Previous studies have indicated that L4 inhibitory neurons receive both thalamocortical^23^,^24^ and local excitatory input^25^,^26^,^27^. However, in our study, the large jitter and variability of excitatory cortical neurons do not affect the reliability of inhibitory neurons in the auditory cortex. One possible explanation is that thalamic input is dominant for cortical inhibitory neurons. An *in vitro* study showed that thalamic input to cortical inhibitory neurons is about 3 times stronger than that to excitatory neurons^27^. Another possible explanation is that input-output relations of inhibitory neurons may vary from excitatory neurons. Previous studies suggested that fast-spiking neurons may have different electrophysiological properties compared with excitatory neurons^28^,^29^. Yet this remains an open question and may be solved by *in vivo* intracellular recording from inhibitory cells.

Also, please note that due to the effects of anesthesia, the contribution of intracortical circuits to spiking variation could have been underestimated in this study. Data obtained from awake and behaving animals should be able to provide more valuable information about variation of neuron activities but could also be more technical demanding. Proper experiment design and data analysis would be essential to justify the results, as many other factors in behaving animals could affect neuron activities such as attention^30^, cortical up-down states^31^, and cross-modal interactions^32^, etc.

As mentioned in the introduction, the role of response variation in the nervous system remains controversial. In this study, we determined the frequency selectivity of excitatory pyramidal neurons and inhibitory interneurons in rat A1. While the response reliability of excitatory neurons was well tuned to sound frequency, their temporal jitter was poorly selective. On the other hand, the weak frequency selectivity of the inhibitory neurons could be attributed to their highly reliable responses. This suggested that the response variation in cortical neurons is not just biological noise but could be a strategy for efficient coding (e.g. sparse coding), because the cortex does have the capability to generate highly reliable responses. Our results revealed an inherent cortical synaptic contribution to the generation of variation in the spike responses of auditory cortical neurons. Because the spike variation of the excitatory neurons was stimulus dependent, manipulation of the variation through the properties of synaptic circuitry may create a new dimension for information coding and processing.

## Materials and Methods

### Animal preparation

All experimental procedures were approved by the Animal Care and Use Committee of Third Military Medical University. All methods were carried out in accordance with the approved guidelines. Experiments were performed in a double-shielded soundproof booth (Shenyang Sound-Proof Booth Factory, China). Adult female Sprague-Dawley rats (1.5–2 months, 160–200 g) were anesthetized with urethane (1.5 g/kg, intraperitoneal injection), whisker shaved and then head fixed by a customized apparatus that allowed the delivery of free-field sound. Because this study required relatively long recording time due to the large number of repetitions, urethane was chosen to minimize the influence of fluctuating anesthesia levels due to its stable and long-lasting effects. To better justify the results, for a small number of control experiments, animals were anesthetized with different anesthesia methods (ketamine + xylazine mixture: ketamine 55 mg/kg, xylazine, 6.4 mg/kg; Nembutal: 60 mg/kg). All presented data and statistics are based on recordings from experiments carried under urethane anesthesia unless otherwise specified. The right auditory cortex was exposed by craniotomy and durotomy. The ear canal on the same side was plugged with a cotton ball. Body temperature was monitored and maintained at 37 °C using a customized heating pad with feedback circuit. All recordings were performed with lights turned off.

### Sound generation and calibration

A free-field magnetic speaker (MF1, TDT Inc., USA) was placed 1 cm away from the left ear of the animal. The speaker was driven by a stereo power amplifier (SA1, TDT Inc., USA) and calibrated using a 1/4″ pressure microphone setup with a prepolarized condenser (377A01 microphone + 426B03 preamplifier + 480E09 signal conditioner, Piezotronics Inc., USA). The captured signals were sampled at 1 MHz/s by a high-speed DAQ board (PCI-6251, National Instruments, USA). Customized LabVIEW programs were used for calibration and sound generation.

### *In vivo* extracellular multiunit recording

Extracellular multiunit recording using parylene-coated tungsten electrodes was performed (0.1 MΩ, WPI Inc., USA) to localize A1 based on the anatomy of the rat brain and the tonotopic gradient^33^,^34^. The electrode was vertically inserted into the cortex and lowered to the thalamocortical layer^16^,^35^,^36^, at a depth of 450–580 μm (layer 4) below the pia, by a powered micromanipulator capable of tracking depth (DMA-1511, Narishige, Japan). A total of 568 pure tones (0.5–64 kHz at 0.1-octave steps; 0–70 dB at 10 dB steps; 35 ms duration; 5 ms sine ramp; 250 ms interstimulus interval) were delivered to the left ear in pseudo random order (to avoid two-tone inhibition and adaptation) and considered as one trial. Neural signals were amplified and collected by a TDT System 3 platform (Gain: 20000, sampling rate: 50 kHz, TDT Inc., USA). The high/low pass filters were set at 300/3000 Hz for the recording of spike activity, and the threshold for spike detection was set at 3 times the standard deviation above baseline. The multiunit spike rate and spike timing were automatically analyzed online and stored for offline analysis by Brainware (TDT Inc., USA).

### *In vivo* patch-clamp recording

#### Loose patch recording

After the localization and mapping of A1, single neuron spike responses were obtained by loose-patch recording^15^,^37^,^38^. A glass pipette (impedance: 5–7 MΩ, prepared by a micropipette puller, P-1000, Sutter Inc., USA) filled with artificial cerebrospinal fluid (ACSF, in mM: 124 NaCl, 1.23 NaH_2_PO_4_, 2.6 NaHCO_3_, 3 KCl, 2 CaCl_2_, 2 MgCl_2_, 10 glucose, 2% biocytin, pH 7.25) was vertically inserted into A1 by a powered micromanipulator (DMA-1511, Narishige, Japan). Mild positive pressure was applied during penetration to keep the pipette tip clean. The pressure was released to form a loose patch when the pipette tip hit a neuron which can be recognized by an increase in resistance and the presence of tiny spikes. Warm agarose dissolved in ACSF (2.5%, 37 °C) was used to cover the recording window to minimize pulsation. Extracellular signals were recorded and amplified by an EPC -10 patch-clamp amplifier (HEKA Gmbh, Germany) in voltage-clamp mode. The cell type of the recorded neuron was initially determined based on the trough-to-peak interval of the spike waveform and then further confirmed by biocytin-based histological reconstruction after the recording session^39^,^40^ (See Histology for details). The typical trough-to-peak interval of excitatory neurons was longer than 0.5 ms. For inhibitory interneurons, this interval was shorter than 0.4 ms^10^,^41^. For the analysis of loose-patch recording data, only data from experiments with at least 10 repetitions were included.

#### Whole-cell recording

Whole-cell recordings were obtained from neurons located 450–590 mm below the pia, which corresponds to layer 4 of rat auditory cortex. Warm agarose dissolved in ACSF (2.5%, 37 °C) was used to cover the recording window to minimize pulsation. For current-clamp recordings, the internal solution contained (in mM) 125 K-gluconate, 4 MgATP, 0.3 GTP, 10 phosphocreatine, 10 HEPES, 1 EGTA, and 1% biocytin (pH 7.2). For voltage-clamp recordings, the internal solution contained (in mM) 125 Cs-gluconate, 5 TEA-Cl, 4 MgATP, 0.3 GTP, 10 phosphocreatine, 10 HEPES, 1 EGTA, 2 CsCl, 1.5 QX-314, and 1% biocytin (pH 7.2). The pipette and whole-cell capacitance were completely compensated, and the series resistance (20–50 MΩ) was compensated by 45–50% to achieve an effective series resistance between 10 and 25 MΩ. Signals were filtered at 2.9 kHz through a low-pass Bessel filter and sampled at 10 kHz (EPC 10, HEKA Gmbh, Germany). To record tone-evoked synaptic conductance, neuronal membrane potentials were clamped at −70 mV and 10 mV for excitatory and inhibitory currents, respectively. The quality of voltage-clamp in our recordings was reasonably good, as demonstrated by the linear I-V relationship of the recorded synaptic currents (Fig. 2B). Recordings in which the neuronal resting membrane potential was more negative than −55 mV and the series resistance was stable (<20% change during the course of the recording session) were used for further analysis. The onset of excitatory and inhibitory currents were measured as the time point at which the response amplitude exceeded 3 times the SD of the base line. As previously reported and discussed^10^,^14^, our whole-cell recordings (which are performed using pipettes with a large tip size) can exclusively target excitatory pyramidal neurons, which has been further confirmed by morphological reconstruction of neurons post-recording. The morphological details of 50% recorded neurons were reconstructed and all the identification results were matched with the results based on spike shapes. For the analysis of whole-cell recording data, only the data from experiments with at least 3 repeated trials were included. Although previous studies have suggested that the space clamping issue and cable effect could result in underestimated measurement of synaptic conductance^11^,^40^, our conclusions are based on the relative change or fluctuation of synaptic conductance. Besides the relative timing between different synaptic inputs is not significantly affected as indicated by previous work using *in vivo* patch clamp approach^11^.

### Histology

Rats were deeply anesthetized with urethane (i.p.) after the recording session and perfused transcardially with saline and 4% paraformaldehyde (dissolved in phosphate-buffered saline, PBS). After decapitation, the brain was transferred to 4% paraformaldehyde and post-fixed for at least 24 hours at 4 °C. Then, the brain was sectioned into 200 μm transverse sections using a vibratome. The sections were rinsed in PBS, and peroxidase activity was blocked by hydrogen peroxide (1%). The sections were then incubated with an avidin–biotin HRP complex (ABC Kit, Vector Laboratories, Inc., USA) at 37 °C for 2 hours. After the sections were thoroughly rinsed in PBS, they were transferred into DAB solution(Vector^®^ SG Subtrate Kit, Vector Laboratories, Inc., USA), and the immunoreaction was monitored under a microscope. Then, the sections were embedded. After reconstruction, the recorded neuron can be classified based on the pyramidal-shaped soma and the apical dendrite^39^,^42^.

### Data analysis

Online analysis was performed using commercial software: Brainware (TDT Inc., USA) for extracellular recording and Patch Master (HEKA Gmbh, Germany) for *in vivo* patch-clamp recording. Offline analysis was performed using customized scripts in Matlab (MathWorks Inc., USA), and SPSS (IBM Inc., USA) was used for statistical analysis. For the loose-patch recordings, the evoked spike response was defined as the spikes that occurred within a time window defined by the average PSTH over all trials (peak ± 10 ms). Failure rate(FR) was defined as the ratio between the number of trials that failed to generate a spike and the total number of trials. First spike jitter was calculated as the standard deviation of the first spike latency across different trials. Experimental data recorded using *in vivo* current-clamp were filtered through a 5 ms median filter to remove spikes to obtain the evoked postsynaptic potential (ΔPSP). Integration time was defined as the interval between the onset of depolarization and the time of the spike activity. For the comparison of Pearson correlation coefficients between different groups, Fisher’s Z-transformation was performed first.

### Synaptic conductance

Excitatory and inhibitory synaptic conductance were derived according to previous reports^10^,^43^,^44^:


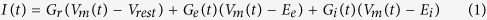


I(t) is the amplitude of synaptic current at time point t. G_r_ represents the resting conductance, and Vrest is the resting membrane potential. Both of these measures were derived from the baseline current prior to each sound stimulation^45^. V_m_ is the holding voltage. G_e_ and G_i_ are the excitatory and inhibitory conductance, respectively. E_e_ (0 mV) and E_i_ (−80 mV) are the reversal potentials for excitation and inhibition, respectively. In this study, V_m_(t) was corrected according to V_m_(t) = V_hold_–R_s_ × I(t), where V_hold_ was the holding voltage and R_s_ was the effective series resistance. A junction potential of approximately 11 mV was corrected. Then, G_e_ and G_i_ could be calculated from this equation by holding the cell membrane at E_e_ and E_i,_ respectively. G_e_ and Gi embody the pure excitatory and inhibitory synaptic input strength.

It should be noted that the derivation of synaptic conductance was based on the assumption that the recorded neurons were linear and isopotential, which could lead to underestimation of absolute synaptic conductance amplitude but not of the relative time^11^. We minimized these absolute errors by comparing conductance across trials within a single cell. Additionally, in the computational model, we simulated membrane potential responses based on a larger range of randomly generated excitation and inhibition, which could further reduce the underestimation of conductance amplitude.

### Modeling

Both excitatory and inhibitory synaptic inputs were generated based on an alpha function that enabled control of the amplitude and temporal profiles^46^:


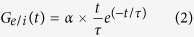


where α and τ represent the amplitude and time constant of the simulated synaptic conductance, respectively. G_e/i_ is the simulated excitatory or inhibitory conductance at time point t. To estimate spike responses, we used an integrate-and-fire model to simulate the membrane potential responses based on synaptic conductance^11^:


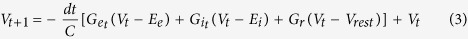


The values of G_r_ (15 nS) and C (100–150 pF) were based on measurements made during the experiments^45^. The resting membrane potential (Vrest) was set to −70 mV, and the action potential threshold was set to 15 mV above E_r_. Based on the binary spiking property of primary cells in the auditory cortex^13^, we only counted the first spike of each simulated trial. Post-synaptic potentials (PSPs) were then simulated by the integrate-and-fire model. The PSPs were simulated from excitatory and inhibitory inputs with random α and τ. After 1 million repetitions, the simulated spike results (1 million) were clustered into groups based on the amplitude and temporal profile of the synaptic inputs. Spike variation and temporal jitter were then calculated within groups with similar input parameters.

## Additional Information

**How to cite this article**: Tao, C. *et al*. Synaptic Basis for the Generation of Response Variation in Auditory Cortex. *Sci. Rep.* **6**, 31024; doi: 10.1038/srep31024 (2016).

## Supplementary Material

Supplementary Information
