# Supplementary material for: Synaptic Basis for the Generation of Response Variation in Auditory Cortex
Source: Sci Rep. 2016 Aug 3;6:31024. doi: 10.1038/srep31024 (PMC4971572; doi:10.1038/srep31024)
Supplement: Supplementary Information [file srep31024-s1.doc]

Synaptic Basis for the Generation of Response Variation in Auditory Cortex

Can Tao^1†^, Guangwei Zhang^1†^, Chang Zhou^1^, Lijuan Wang^1^, Sumei Yan^1^,

Li I. Zhang^2^, Yi Zhou^1*^, Ying Xiong^1*^

1. Department of Neurobiology, College of Basic Medical Sciences, Third Military Medical University, 30 Gaotanyan St., Chongqing, 400038, China

2. Zilkha Neurogenetic Institute, Keck School of Medicine, University of Southern California, Los Angeles, California 90033, USA

^†^ These authors contributed equally to this work.

Address correspondence to Y. Zhou ([zhouyisjtu@gmail.com](mailto:zhouyisjtu@gmail.com)) or Y. Xiong ([xiongying2001@163.com](mailto:xiongying2001@163.com)), Department of Neurobiology, College of Basic Medical Sciences, Third Military Medical University, Chongqing, China


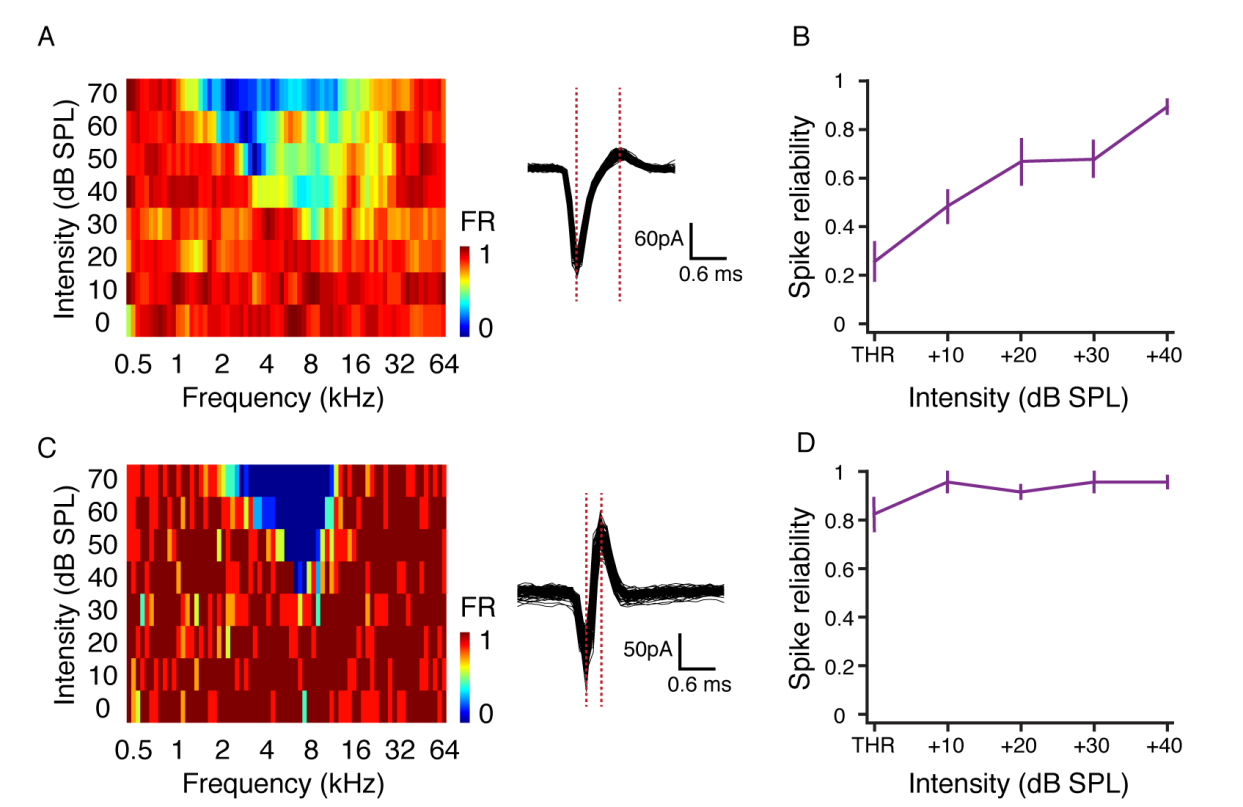
Supplementary Figure 1

(**a**). An example TRF of failure rate from an excitatory neuron in layer 4 of the primary auditory cortex. Color represent failure rate to certain stimulus. Right panel showed overlapped spike waveforms (n = 50). Red dotted lines indicate the trough-to-peak interval.

(**b**). Relationship between spike reliability and sound intensity of excitatory neurons, n=27. Error bar, SE.

(**c**). An example TRF of failure rate from an inhibitory neuron in layer 4 of the primary auditory cortex. Color represent failure rate to certain stimulus. Right panel showed overlapped spike waveforms (n = 50). Red dotted lines indicate the trough-to-peak interval.

(**d**). Relationship between spike reliability and sound intensity of inhibitory neurons, n = 9. Error bar, SE.


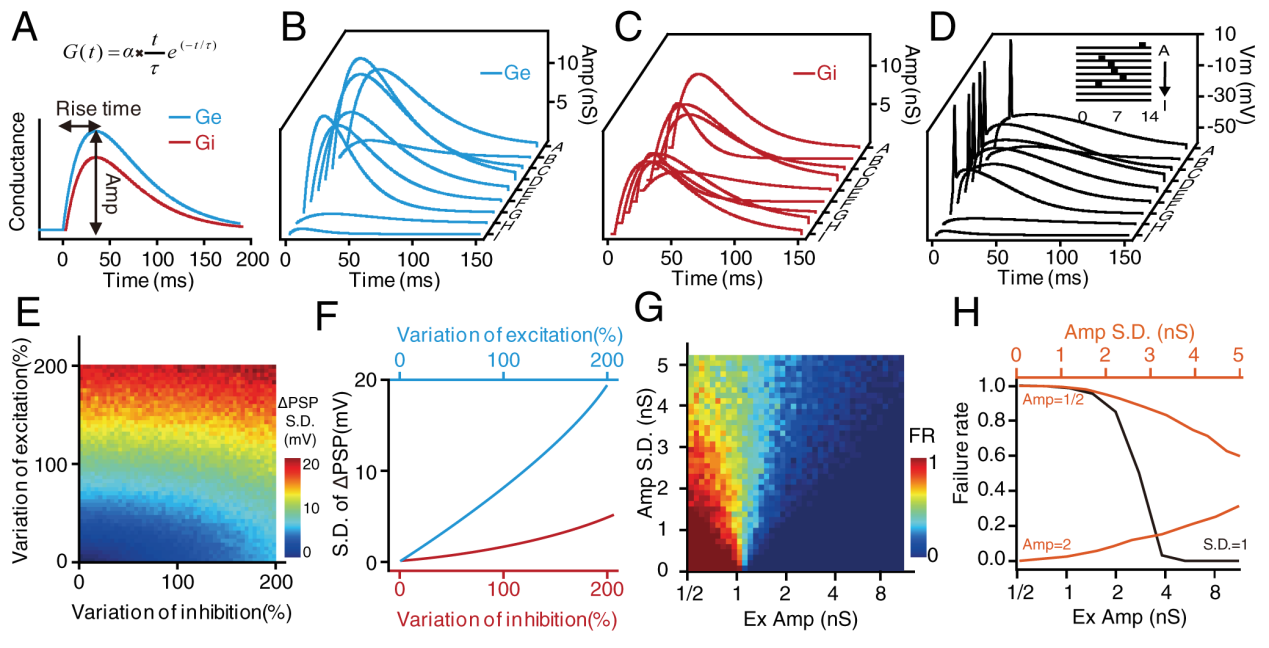
Supplementary Figure 2. Modeling the contribution of synaptic inputs to failure rate

(**a**) Example of the excitatory (blue) and inhibitory (red) conductance generated by the alpha function. (**b-c**) Nine examples of simulated excitatory (b) and inhibitory(c) conductance as well as the integrated membrane potential responses. (**d**) The simulated membrane potential change based on the examples shown in **b&c**. In the inset panel, the dots represent the simulated action potential responses. (**e**) The contribution of fluctuation in excitation and inhibition to the variation (S.D.) of evoked postsynaptic potential (ΔPSP). (**f**) The contribution of fluctuation in excitation or inhibition to the variation (S.D.) of the evoked membrane potential when the other component was fixed. Blue, with fixed inhibition. Red, with fixed excitation. (**g**) The contribution of the absolute amplitude as well as variation in the amplitude (S.D.) of excitatory synaptic inputs to the FR. Color represents the level of FR. (**h**) The contribution of the absolute amplitude as well as variation in the amplitude (S.D.) of excitatory synaptic inputs to the FR when the other component is fixed. Black, with fixed amplitude variation (S.D. = 1 nS). Orange, with fixed amplitude (Amp = 1/2 or 2 nS).

Because the ΔPSP is critical for the generation of spike activity (Fig. 1H&I), we first estimated the contribution of fluctuation in the amplitude of excitation and inhibition to the change in ΔPSP. The amplitudes of the synaptic conductance were drawn from a Gaussian distribution (10 ± 10 nS, mean ± S.D.) for both excitatory and inhibitory inputs (Fig.S2E). The onset latency of excitation was set to be 2 ms earlier than that of inhibition, in accordance with the experimental data. When the variation in the amplitude of excitation doubles, the S.D. of the ΔPSP increased by 19.6 mV; when the variation in the amplitude of inhibition doubled, the S.D. of the ΔPSP increased 5.4 mV (Fig. S2F). This result suggested that the ΔPSP is more sensitive to variation in the amplitude of excitatory inputs than in inhibitory inputs. Considering that the amplitude of synaptic inhibition is also more stable than that of excitation, we next focused on the contribution of fluctuation in excitation to the FR (Fig. S2G). Based on the experimental data, the amplitude of inhibitory input was set to be 4 nS on average, with a standard deviation of 1 nS; the amplitude of excitatory inputs varied within the range of 0.5 to 10 nS, with a standard deviation within the range of 0 to 5.5 nS. When the amplitude was relatively large (> 2 nS), increasing the variation in amplitude resulted in a larger FR (Fig. S2H, Amp = 2). On the other hand, when the amplitude was relatively small (<1 nS), increased variation could reduce the FR (Fig. S2H, Amp = 1/2). Meanwhile, increasing the amplitude of the excitatory synaptic inputs could monotonically decrease the FR (Fig. S2H, S.D. = 1).


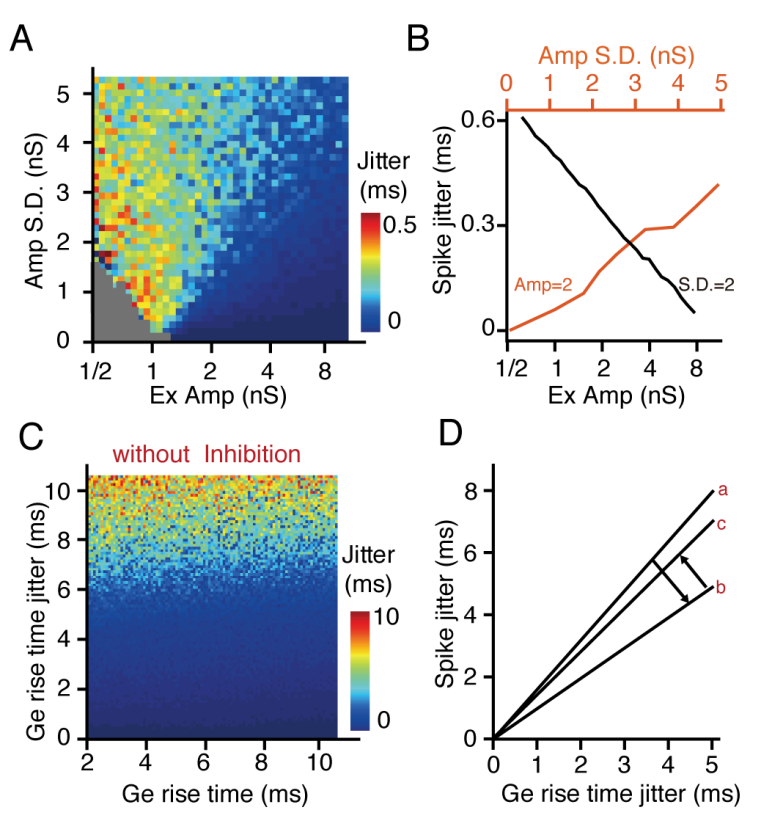


Supplementary Figure 3. Modeling the contribution of synaptic inputs to temporal variation

(**a**) The contribution of the amplitude (Ex amp) and variation (Amp S.D.) of excitatory inputs to 1st spike jitter. The grey area indicates cases of spike failure.

(**b**) The contribution of the amplitude (Ex amp) and variation (Amp S.D.) of excitatory inputs to 1st spike jitter when the other component is fixed. Black, amplitude S.D. = 2 nS; brown, amplitude = 2 nS. (**c**) The contribution of excitatory conductance rise time and rise time jitter to 1st spike jitter when inhibition is omitted. (**d**) The contribution of jitter in the rise time of excitatory inputs to 1st spike jitter without inhibition (line a), with temporally stable inhibition (line b) or with temporally unstable inhibition (line c). a, without inhibition; b, inhibitory amplitude = 4 nS, S.D. = 1 nS, rise time jitter = 0 ms; c, inhibitory amplitude = 4 nS, S.D. = 1 nS, rise time jitter = 4 ms.

Over a large range of amplitudes and variation in the amplitude of excitatory synaptic inputs, only a small change (<0.5 ms) in 1st spike jitter was observed (Fig. S3A&B), suggesting that variation in the amplitude of synaptic inputs could only partially explain the temporal jitter observed in the spike responses. To better understand the underlying mechanism, the contribution of the temporal variation of synaptic inputs was investigated (Fig. S3C). The contribution of the rise time jitter of excitatory inputs was compared to that of 1st spike jitter without inhibition, with temporally stable inhibition or with temporally unstable inhibition (Fig. S3D). Inhibitory inputs were found to be capable of reducing the temporal jitter of the spike response by limiting the potential window of spike activity. However, variation in the rise time of inhibitory inputs could also increase the temporal jitter of spike responses (Fig. S3D).
